# The Significance of Fibrosis Quantification as a Marker in Assessing Pseudo-Capsule Status and Clear Cell Renal Cell Carcinoma Prognosis

**DOI:** 10.3390/diagnostics10110895

**Published:** 2020-11-02

**Authors:** Caipeng Qin, Huaqi Yin, Huixin Liu, Feng Liu, Yiqing Du, Tao Xu

**Affiliations:** 1 Department of Urology, Peking University People’s Hospital, No. 11, Xi Zhi Men Nan Street, Beijing 100044, China; qincaipeng1986@126.com (C.Q.); duyiqing@bjmu.edu.cn (Y.D.); 2 Department of Urology, Henan Provincial People’s Hospital, No. 7, Wei Wu Road, Zhengzhou 450003, China; yhq901212@163.com; 3 Medical Records Statistics Office, Peking University People’s Hospital, No. 11, Xi Zhi Men Nan Street, Beijing 100044, China; lhxepi@163.com; 4 Peking University Hepatology Institute, Peking University People’s Hospital, No. 11, Xi Zhi Men Nan Street, Beijing 100044, China; liu1116m@sina.com

**Keywords:** ccRCC, fibrosis, pseudo-capsule, invasion, progression-free survival

## Abstract

Fibrosis plays an important role in tumor growth and progression, and thus, we aimed to determine whether renal fibrosis is correlated with the clinical and pathological characteristics and prognosis of clear cell renal cell carcinoma (ccRCC). Fibrosis, including intra-tumoral fibrosis (ITF), pseudo-capsule (PC) fibrosis and adjacent normal renal interstitial fibrosis, was evaluated in 73 pairs of ccRCC specimens using second harmonic generation combined with two-photon excitation fluorescence (SHG/TPEF). The clinical and pathological characteristics of the patients who were eligible for the present study were recorded. The associations between fibrosis and clinicopathological parameters were analyzed using a Mann-Whitney U test or logistic regression analysis. Progression-free survival (PFS) was analyzed using the Kaplan-Meier method and a Cox regression model. High-resolution images of fibrosis were captured from unstained slides using the SHG/TPEF approach. Both ITF and PC fibrosis were associated with tumor progression in ccRCC. Multivariate logistic regression analysis revealed a significant inverse association between the PC collagen proportional area (CPA) and PC invasion (*p* < 0.05), suggesting that PC CPA is an independent risk factor or marker for PC invasion. A significant decrease in progression-free survival (PFS), determined by Kaplan-Meier curves, was observed for patients with higher PC CPA status compared with those with lower PC CPA status (*p* < 0.05). Similar results were observed in patients with PC invasion. In multivariate Cox regression analysis, PC invasion and intra-tumoral necrosis were identified as independent prognostic factors for PFS. Our data suggest that ITF and PC fibrosis are associated with ccRCC progression. In addition, PC fibrosis may act as a marker of PC invasion and an effective quantitative measurement for assessing prognosis.

## 1. Introduction

Fibrotic responses are common characteristics of many malignancies, such as prostate cancer, breast cancer and pancreatic adenocarcinoma [1,2,3]. Fibrotic responses in cancer are thought partly to result from the role of activated myofibroblasts or fibroblasts in producing extracellular matrix, and these responses have been suggested to polarize tumor immunity. We hypothesized that tumor-associated fibrosis might serve as an indicator of the different immune statuses of a malignancy. Therefore, a better understanding of fibrosis in malignancies may provide new evidence for preventing tumor progression and exploring potential therapeutic strategies.

Clear cell renal cell carcinoma (ccRCC) is the most common phenotype of renal cell carcinoma (RCC), which is derived from renal tubule epithelial cells and is often characterized by intra-tumoral fibrosis (ITF) and peritumoral pseudo-capsules (PCs). ITF is routinely characterized by deposition of collagen proteins around fibroblasts. In breast cancer, ITF is associated with tumor type and lower lymphocytic infiltration. Therefore, ITF has been identified as having prognostic implications and is correlated with intra-tumoral necrosis and inflammation [4]. In ccRCC, ITF was confirmed to be related to adverse pathological parameters, such as Fuhrman nuclear grade, intra-tumoral necrosis and inflammation, but ITF itself did not have prognostic significance [5]; thus, whether ITF can serve as an indicator of immune status and prognosis in ccRCC should be explored.

PCs are a common pathological characteristic observed during ccRCC development. Previous studies have demonstrated that PC status has good prognostic implications in RCC [6]. The disappearance or thinning of PCs has been associated with nuclear grade, TNM stage, tumor size and necrosis and suggests poor prognosis of patients with RCC [6,7]. As a fibrous capsule might indicate the immune status surrounding a tumor, quantification of the fibrosis in PCs might be useful for assessing PC status and patient prognosis. Traditionally, fibrosis has been quantified using biochemical and histological methods, such as Masson’s trichrome staining, picrosirius red staining and immunohistochemistry (IHC), but these methods are limited to assessing fibrosis based on variations in staining and visual assessment [8,9].

Currently, tissue microarchitecture and inflammatory infiltrates can be analyzed by using whole-slide or state-of-the-art nonlinear image analysis techniques, such as fractal dimension and sample entropy of internuclear distances [10,11]. In the present study, we quantified fibrosis in ccRCC using an approach combining second harmonic generation with two-photon excitation fluorescence (SHG/TPEF); with this approach, fibrosis in ccRCC tissues was clearly and quantitatively recorded. We also determined the significance of measuring fibrosis in evaluating PC status and ccRCC prognosis.

## 2. Materials and Methods

### 2.1. Patients and Tumor Sample Selection

**Figure 1:**
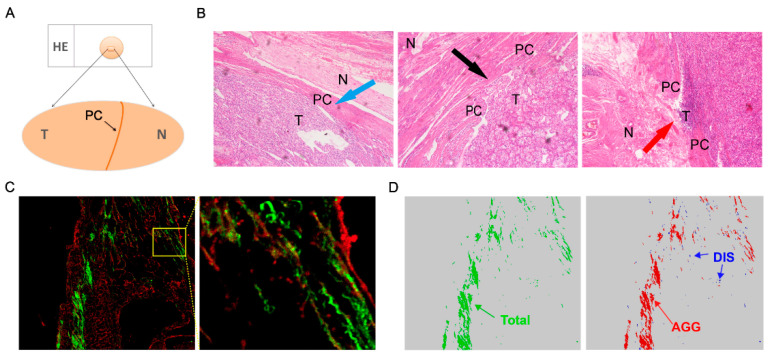
Acquisition of paraffin sections and illustration of PC status and parameters. (**A**) Paraffin sections containing tumor, PC and adjacent normal parenchyma tissues were identified according to HE staining (Hematoxylin eosin staining). (**B**) PC status: PC-, completely intact PC (blue arrow); PC+, PC invasion with (black arrow) or without tumor penetration (red arrow). (**C**) Illustration of collagen proportional area (CPA) (green)/two-photon excitation fluorescence (TPEF) (red) images. (**D**) Illustration of total (green), aggregated (red) and distributed (blue) collagen fibers (T: Tumor; N: Normal tissue; PC: pseudo-capsule; Dis (DisCPA): percentage of distributed collagen proportional area; Agg (AggCPA): percentage of aggregated collagen proportional area).

Data were collected from patients who underwent radical or partial nephrectomy for ccRCC at Peking University People’s Hospital between November 2012 and May 2015 according to a protocol that was approved by the Peking University Institutional Review Board. Patients with the nonclear cell RCC type were excluded. Paraffin sections containing peritumoral PCs accompanied by primary tumor tissues and adjacent renal parenchyma were identified by an experienced pathologist who reviewed HE-stained (Hematoxylin eosin stained) sections from archived paraffin-embedded tissues (Figure 1A). Finally, paraffin sections from 73 patients were identified as being eligible for analysis. The demographic, clinical and pathological data and other characteristics of the patients who were eligible for the present study were gathered from their electronic medical records and are summarized in Table 1. PC status was carefully assessed by an experienced genitourinary pathologist and classified as follows (Figure 1B): PC-, the PC around the tumor is unbroken and continuous; PC+, tumor cells invade into or out of the PC [6]. All the patients were followed up every six months. The last follow-up was 25 September 2019.

### 2.2. SHG and TPEF Imaging

Tissue fibrosis was quantified using images acquired from the Genesis (HistoIndex, Singapore) system, and this quantification included the SHG and TPEF techniques. TPEF relies on nonlinear light–matter interactions to provide contrast and optical sectioning capability for high-resolution imaging. SHG from structural proteins has emerged as an important new contrast mechanism. SHG/TPEF has been successfully applied to assess fibrosis in several tissues and organs, including the kidney. Compared with traditional staining, for example Masson’s trichrome and Sirius red staining, SHG/TPEF is a sensitive, quantitative, and automated tool that defines collagen in fibrotic tissues. Briefly, unstained paraffin sections were initially excited with a 780 nm laser, and then, the SHG signal at 390 nm and the TPEF signal at 550 nm were recorded. The SHG signal (green) is sensitive to the non-centrosymmetric microstructure of the tissues and has been successfully applied to evaluate fibrosis in many organs, including the kidney [12]. The TPEF signal (red) is suitable for imaging cell and tissue morphology after tissue samples are stained with endogenous or exogenous fluorescent dyes. As shown in Figure 1C, fibrosis was identified by the SHG signal, and the organizational structure of the tissue samples was identified by the TPEF signal. Therefore, images using SHG combined with TPEF (SHG/TPEF) are suitable for visualizing the fibrosis and organizational structure of tissue samples and for obtaining the collagen proportional area (CPA), calculated as SHG value/(SHG value + TPEF value).

### 2.3. Quantification of Collagen Morphology Parameters

To evaluate fibrosis, selected paraffin sections were subjected to SHG and TPEF microscopy. The CPA can be categorized into the aggregated collagen proportional area (AggCPA) and the distributed collagen proportional area (DisCPA) based on collagen crosslinking properties. In the present study, fibrosis in the samples was primarily measured by assessing three collagen patterns: total collagen (CPA), aggregated collagen (AggCPA) and distributed collagen (DisCPA) (Figure 1D). In addition, 32 collagen morphological parameters were extracted, including the percentage of collagen and the features of fibers. For a single collagen fiber, the collagen fiber percentage, width, length, perimeter and other parameters are shown in supplementary Figure S1. For the total collagen pattern, the morphological parameter listed as the percentage of total collagen was unique, and the morphological parameter listed as the number of cross links (NoXlinks) belonged to the pattern of aggregated collagen. The other 10 morphological parameters were shared by the three collagen fiber patterns. In the current study, we focused mainly on the CPA, AggCPA, and DisCPA (Figure 1D). All the signals of the parameters were normalized to the TPEF signals before being subjected to analysis.

### 2.4. Statistical Analysis

Statistical analysis was performed using SPSS 19.0 (SPSS Inc., Chicago, IL, USA) or GraphPad Prism 5.0. Differences in fibrosis, presented as the median ± IQR between two groups, were analyzed using the Mann-Whitney U test. Logistic regression analysis was used to analyze associations between PC status and clinicopathological features or fibrosis. Progression-free survival (PFS) was plotted using the Kaplan-Meier method, and differences between different groups were determined with a log-rank test. Cox’s proportional hazards model was applied in univariable or multivariable analysis to assess correlations between prognosis and fibrosis and other parameters. *p* < 0.05 was considered to be significant.

## 3. Results

### 3.1. Association between ITF and Clinicopathological Characteristics

**Figure 2:**
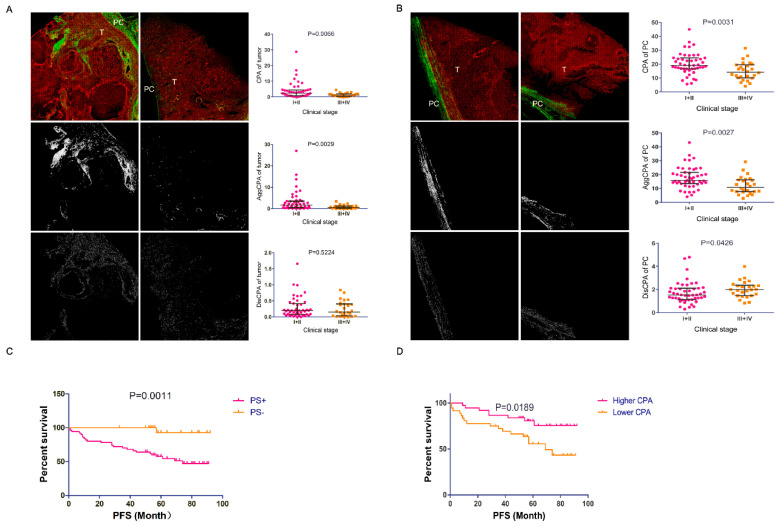
Quantification and significance of fibrosis. Representative images of second harmonic generation (SHG), AggCPA and DisCPA in tumors (**A**) and PCs (**B**). (**C**) Kaplan-Meier curves of progression-free survival (PFS) of patients with PC+ and PC- status. (**D**) Kaplan-Meier curves of PFS of patients with higher or lower PC CPA status (T: tumor; PC: pseudo-capsule; CPA: collagen proportional area; Dis (DisCPA): percentage of distributed collagen proportional area; Agg (AggCPA): percentage of aggregated collagen proportional area).

On the basis of SHG, 31 out of 73 (42.5%) patients had fibrosis in the adjacent renal parenchyma, while 95.9% and 100% of patients had intratumor and PC capture, respectively (*p* < 0.001). To investigate the impact of fibrosis on ccRCC, we initially analyzed the three ITF patterns. We observed differences in the intra-tumoral CPA between low clinical stage (I + II) and high clinical stage (III + IV), demonstrated by the fact that higher intra-tumoral SHG signals were observed in the low-grade samples than in the high-grade samples (Figure 2A). Next, we analyzed the AggCPA and DisCPA in the tumors. In the low-stage samples, the median intra-tumoral AggCPA was higher than in the high-grade samples (Figure 2A). However, no differences in the intra-tumoral DisCPA were detected between the low- and high-stage tumors (Figure 2A). Next, we analyzed differences in fibrosis between the different groups classified by other clinicopathological parameters. As shown in supplementary Table S1, intra-tumoral DisCPA was increased in tumors with a larger size (*p* = 0.007) and intra-tumoral necrosis (*p* = 0.030). No association was found between the three fibrosis patterns and other clinicopathological characteristics. These results demonstrate the possibility that ITF was destroyed in the process of ccRCC progression, indicating a decreased barrier for cancer cell motility.

### 3.2. Association between PC Fibrosis and Clinicopathological Characteristics

Subsequently, we analyzed the relationship between PC fibrosis and the aforementioned variables, and the results are reported in supplementary Table S2. Both the median CPA and AggCPA in the low clinical stage group were greater than those in the high clinical stage group, while the opposite trend was observed for the DisCPA (Figure 2B). In addition, a greater median DisCPA was observed in patients with high Fuhrman grade (*p* = 0.004) and tumor size (*p* = 0.044). Remarkably, the CPA (*p* = 0.029) and AggCPA (*p* = 0.043) detected in patients aged ≤58 years were both greater than those in patients aged >58 years, suggesting a correlation between fibrosis and age. No statistically significant differences in the three fibrosis patterns were found among the groups divided by other variables. In summary, these results show that the PC was destroyed during tumor progression, which may increase the risk of PC penetration by cancer cells, allowing invasion of adjacent normal renal parenchyma.

### 3.3. PC fibrosis was a Marker for PC Invasion

We next sought to determine whether the fibrosis detected by the SHG/TPEF approach was correlated with PC status. Of the 73 patients, 50 (68.5%) had PC invasion (PC+), and 23 (31.5%) exhibited an intact PC (PC-). As shown in Table 2, univariate logistic regression analysis revealed a significant association between PC+ status and the CPA and AggCPA in the PC, indicating that PCs with decreased CPA and AggCPA are more easily subject to cancer involvement, while there was no significant correlation between PC+ status and DisCPA in the PC. In addition, PC+ status was meaningfully correlated with clinical stage and tumor size, while there was no association between PC+ status and sex, age, tumor side, intra-tumoral necrosis or the three ITF patterns. However, an increased risk of PC+ status in patients with ≥G2 grade was observed compared to patients with G1 grade, as determined by univariate logistic regression analysis (odds ratio (OR) 3.343; 95% confidence interval (CI) 1.194–9.363; *p* = 0.022). Subsequently, multivariate logistic regression analysis was performed to determine which variables that were significant in the univariate analysis could act as independent predictors of PC status. The PC AggCPA was not included in the multivariate analysis because the percentage of all the AggCPA was approximately equal to the percentage of the CPA. As expected, PC CPA and tumor size were identified as markers of PC invasion. These data suggest that quantification of fibrosis in the PC is of significance in assessing PC status.

### 3.4. The Prognostic Role of PC CPA as a Marker

Prior to the last follow-up, 25 patients had been diagnosed with tumor progression. Among these patients, one was in the 23 PC- cohort, while 24 were in the 50 PC+ cohort. Kaplan-Meier curves showed worse PFS among the PC+ patients than among the PC- patients (log-rank test, *p* = 0.0011; Figure 2C). Using the median as a cutoff, the PC CPA was divided into higher and lower statuses (PC CPA status). The patients with a higher PC CPA had longer PFS than those with a lower PC CPA (log-rank test, *p* = 0.019; Figure 2D), based on Kaplan-Meier curves. In addition, univariate Cox regression analysis demonstrated that PC status, tumor size, Fuhrman grade, intra-tumoral necrosis, PC CPA and AggCPA were significant predictors of PFS. After adjustment via multivariable Cox regression analysis, only intra-tumoral necrosis and PC status served as independent predictors of PFS (Table 3). PC+ patients had an approximately 10-fold increased risk of disease progression compared with PC- patients (HR 10.529; 95% CI 1.362–81.419; *p* = 0.024). However, PC CPA was not identified as an independent prognostic factor for PFS (HR 0.949; 95% CI 0.889–1.013; *p* = 0.116). On the other hand, if we classified PC CPA based on higher and lower status as described above, not only intra-tumoral necrosis and PC status but also PC CPA status had significant prognostic impacts on PFS according to the multivariable Cox regression model (HR 2.715; 95% CI 1.088–6.774; *p* = 0.032); these results suggest that a decreased PC CPA has an adverse impact on PFS. These results demonstrate the potential prognostic role of PC CPA as a marker of PFS.

## 4. Discussion

Fibrosis plays vital roles in tumor growth and progression. Previous studies have demonstrated fibrotic responses in various malignances, including RCC [5,13]. In addition, cancer-associated fibroblasts contribute to cancer proliferation and invasion by secreting certain cellular factors [14]. Fibrosis involves many complex factors, including fibroblast activation, dense collagen deposition, etc., and together with tumor cells, certain immune cells and cytokines, fibrosis contributes to the formation of the tumor microenvironment (TME). Desmoplasia has been hypothesized as affecting immune cell infiltration, including infiltration of both T cells and immune-suppressive cells, such as Treg cells, myeloid-derived suppressor cells and M2 macrophages. For instance, cancer-associated fibroblasts in the TME can impair immunity in esophageal cancer by affecting T leukomonocytes [15,16]. We hypothesize that fibrosis results from the interaction of desmoplasia, tumors and the immune system and thus may act as a parameter for assessing tumor status. Therefore, precise quantification of fibrosis may be helpful for understanding the role of fibrosis in cancer.

In the current study, we demonstrated that fibrosis was a more common phenomenon in intratumor tissues than in adjacent normal tissues in ccRCC, which confirmed the presence of fibrosis in ccRCC as demonstrated by previous studies [5]. Fibrogenesis in intra-tumoral tissues specifically contributes to the formation of a fibrotic TME that may in turn affect the progression of ccRCC itself or the tumor immune microenvironment. In addition, our data revealed that the intra-tumoral CPA and AggCPA in low-grade tumors were higher than those in high-grade tumors, which was in contrast with a previous study that demonstrated a negative correlation between ITF and tumor grade. This difference may be due to the different approaches used to assess fibrosis in our study and previous studies; however, the method we used is more quantifiable. Moreover, our study reduced the impact of subjective factors on the quantification of fibrosis to a greater extent. Furthermore, the intra-tumoral DisCPA was found to have a significant positive correlation with tumor size and histologic necrosis, suggesting destruction of fibrosis in the tumor progression process. These results demonstrate that fibrosis arises with oncogenesis and is decreased in tumor development, which may indicate changes in the TME. The limitation of the current study was the local assessment of fibrosis in only some tumor tissues. Therefore, the overall assessment of fibrosis in larger sections of whole tumor tissues may further reveal the fibrotic status in ccRCC.

As a striking characteristic, the PC in RCC has been studied for a long time due to its prognostic value, and this value has been reported in many studies [17,18,19]. In these studies, PC status was identified as a marker for overall survival or PFS of RCC patients, despite the inconsistent proportions of PC involvement (33% to 72%) [6,7]. Many variables, such as histological type, tumor size, Furman grade, TNM stage, and intra-tumoral histologic necrosis, were verified as being related to PC involvement. Although these factors are risk factors for PC involvement, the existence of individual differences reduces the value of these factors in predicting PC status. In fact, the assessment of PC status is still dependent on pathological evaluation under a microscope by pathologists, which easily results in omissions. PC thickness is an obvious quantitative indicator for evaluating PC status, and a thin PC indicates high risk of cancer penetration [6]. However, when measuring the PC, it is easy to overestimate PC thickness by calculating nonfibrous components and to ignore the complex components of fibrosis that are vital barriers in preventing cancer penetration.

Our data revealed that the PC CPA and AggCPA in low-stage tumors were both higher than those in high-stage tumors, while the DisCPA exhibited the opposite trend. The CPA was the total of the AggCPA and DisCPA. These results demonstrate that the PC CPA decreased with ccRCC progression, which may indicate that the immune response became weaker and that the tumor cells became more aggressive. Based on univariate logistic regression analysis, we confirmed that the ratio of PC invasion increased when the PC CPA and AggCPA decreased, suggesting an inverse association of the CPA or AggCPA with cancer progression. In addition, clinical stage was found to be associated with PC invasion in the univariate logistic regression analysis. The Fuhrman grade reported by Minervini et al. could act as an independent risk factor for PC invasion [7]; however, it was not associated with PC invasion in a recent study conducted by Wei Xi et al. [6]. In our study, if Fuhrman grade was classified into low (G1+2) and high (G3+4), no significant association between Fuhrman grade and PC invasion was detected. However, there was a meaningfully increased risk of PC invasion in tumors with Fuhrman grades > G2 compared with tumors in Fuhrman grade G1 (OR 3.343; 95% CI 1.194–9.363; *p* = 0.022). Multivariable analyses demonstrated that tumor size and PC CPA were independent markers of PC invasion. Although the 4-cm tumor size cutoff could significantly predict PC status, it should still be examined to define PC invasion [7]. Notably, there was no significant association between PC invasion and tumor size if we changed the cutoff from 4 to 7 cm, indicating that there were other factors involved. For instance, a tumor > 7 cm in size could still exhibit a PC if it were surrounded by abundant fibrosis. Therefore, combined quantification of the PC CPA seems to be a better approach for predicting PC status.

In previous studies, certain biomarkers of ccRCC were identified, such as urinary RKIP/p-RKIP, kynurenine-to-tryptophan ratio (KTR), glucose-6-phosphate isomerase (GPI)/autocrine motility factor (AMF), αKlotho, CA 15–3, CA 125, and β-2 micro-globulin, and a few metabolomics-based biomarkers have been reported to be associated with ccRCC prognosis [20,21,22,23,24,25,26,27]. Consistent with previous studies, the current study revealed a significant association between PC status and ccRCC prognosis. Of the 73 patients in our study, 25 (34.2%) died or suffered progression during follow-up. Considering the limitation of death cases, PFS was used as the end point. PC status, intra-tumoral necrosis, tumor size, and PC CPA and AggCPA were identified as predictors of PFS in the univariate Cox regression analysis. However, only PC status and intra-tumoral necrosis were identified as significant independent prognostic factors for PFS in ccRCC patients, which was consistent with the results of a previous study [6]. However, the PC CPA status had an independent prognostic impact on PFS, indicating a potential prognostic impact of the PC CPA on PFS. The insignificance of PC CPA in multivariable Cox regression analysis may have resulted from the uneven distribution of patients in different clinical stages, the small cohort of patients or the short follow-up time in the current study. Inconsistent with previous studies, our study confirmed, for the first time, an association between ccRCC prognosis and nontraditional clinicopathological variables, namely fibrosis.

As a quantitative index, the PC CPA can be measured independently of other related factors, such as PC thickness, and may be a promising potential prognostic biomarker due to its accuracy. Specifically, imaging of the PC CPA can clearly reflect the fibrosis content in the exact region of the PC, which could help reveal the most likely position of PC invasion or penetration. Therefore, patients with a lower PC CPA should be subjected to closer surveillance. In previous studies, researchers evaluated tissue microarchitecture and inflammatory infiltrates in cancer tissue though state-of-the-art nonlinear image analysis techniques or whole-slide images to estimate fractal dimension and sample entropy of internuclear distances [10,11]. To evaluate the relationship more precisely between fibrosis and ccRCC progression in future research, it may be more exact to measure fibrosis dispersion using fractal dimension or entropy to determine parameters such as the length, width and area of the fibrosis. In conclusion, a quantitative index of fibrosis makes it possible for us to precisely estimate fibrosis and its potential clinical value.

Fibrosis occurs intratumorally and in the PC. Fibrosis consists of a mixture of fibroblasts and collagen fibers. Theoretically, the components of intra-tumoral fibrosis and PC fibrosis should be the same. From this perspective, the inverse association between ITF and tumor progression identified in this study, which contrasted with results of other studies, seems tenable. However, the existence of heterogeneity between intra-tumoral and peritumoral regions and the different proportions of fibrotic components in the intra-tumoral and peritumoral regions may open this explanation to debate. Therefore, ITF requires further investigation. Fibrosis has been reported to be associated with tumor immunity [28,29,30]. Specifically, the correlation between cancer-associated fibroblasts and tumor lymphocytic infiltration revealed in various studies suggests that further investigation of fibrosis in tumors is valuable.

Undoubtedly, the PC was decreased in the tumor progression process. Various factors were involved in PC destruction. Epithelial-mesenchymal transition (EMT) may promote degradation of collagen type IV by activating MMPs [31,32] and is one of the main mechanisms by which RCC tumor cells invade [33,34]. However, whether EMT or other mechanisms lead to destruction of the PC must still be determined.

## 5. Conclusion

Our study highlights the role of fibrosis measurements in assessing PC status and predicting ccRCC patient prognosis. However, due to the small sample size and short follow-up time, comprehensively assessing the role of fibrosis measurements in assessing the PC and patient prognosis is difficult. Thus, an investigation with a large sample size and complete information regarding prognostic and other factors is important.

## Figures and Tables

**Table 1:** Characteristics of patients with clear cell renal cell carcinoma (ccRCC).

Features		Number, *n* (*n* = 73)
Gender	Male	54
	Female	19
Age(years)	≤58	39
	>58	34
Fuhrman grade	G1+2	54
	G3+4	19
Clinical stage	I+II	47
	III+IV	26
Tumor size	≤4 cm	47
	>4 cm	26
Intra-tumoral necrosis	Absent	54
	Present	19
PC status	PS-	23
	PS+	50

PC: pseudo-capsule.

**Table 2:** Logistic regression analysis for pseudo-capsule status.

Factors		Univariate Analysis		Multivariate Analysis	
		OR(95% CI)	*p* Value	OR(95% CI)	*p* Value
Gender	male vs. female	0.995 (0.323–3.066)	0.994		
Age (year)	≤58 vs. >58	1.200 (0.444–3.241)	0.719		
Nuclear Grade	G1+2 vs. G3+4	1.400 (0.436–4.499)	0.572	1.019 (0.274–3.782)	0.978
Clinical Stage	I + II vsIII + IV	3.732 (1.108–12.569)	0.034	2.128 (0.563–8.043)	0.265
Tumor Size	≤4 cm vs. >4 cm	5.679 (1.495–21.577)	0.011	6.409 (1.453–28.264)	0.014
Intra-tumoral necrosis	absent vs. present	1.400 (0.436–4.499)	0.572		
Tumor side	left vs. right	0.562 (0.206–1.537)	0.261		
PC	CPA	0.925 (0.863–0.992)	0.028	0.923 (0.854–0.998)	0.044
	AggCPA	0.925 (0.864–0.991)	0.026		
	DisCPA	1.116 (0.620–2.008)	0.714		
Tumor	CPA	0.942 (0.845–1.051)	0.285		
	AggCPA	0.942 (0.840–1.057)	0.312		
	DisCPA	0.399 (0.078–2.026)	0.268		

OR: Odds Ratio; CI: confidence interval, PC: pseudo-capsule, CPA: collagen proportional area, DisCPA: percentage of distributed collagen proportional area, AggCPA: percentage of aggregated collagen proportional area.

**Table 3:** COX regression analysis for progression free survival (PFS).

Factors		Univariate Analysis		Multivariate Analysis	
		HR (95% CI)	*p* Value	HR (95% CI)	*p* Value
Gender	male vs. female	0.493 (0.169–1.435)	0.194		
Age (year)	≤58 vs. >58	0.943 (0.428–2.078)	0.884		
Fuhrman Grade	G1+2 vs. G2+4	1.532 (0.660–3.588)	0.321	1.259 (0.526–3.013)	0.605
Clinical Stage	I+II vs III + IV	2.036 (0.926–4.475)	0.077	0.823 (0.324–2.093)	0.682
Tumor Size	≤4 cm vs. >4 cm	2.562 (1.159–5.662)	0.020	1.462 (0.608–3.512)	0.396
Tumor side	left vs. right	0.634 (0.273–1.470)	0.288		
Histologic Necrosis	Absent vs. present	2.781 (1.259–6.142)	0.011	2.858 (1.084–7.533)	0.034
PS status	PS- vs. PS+	12.957 (1.751–95.869)	0.012	10.529 (1.362–81.419)	0.024
	CPA	0.938 (0.883–0.996)	0.037	0.949 (0.889–1.013)	0.116
PS	AggCPA	0.928 (0.870–0.991)	0.025		
	DisCPA	1.449 (0.948–2.213)	0.087		
	CPA	1.025 (0.939–1.120)	0.577		
Tumor	AggCPA	1.027 (0.935–1.127)	0.578		
	DisCPA	1.470 (0.411–5.258)	0.554		

HR: hazard ratio, PC: pseudo-capsule, CPA: collagen proportional area, DisCPA: percentage of distributed collagen proportional area, AggCPA: percentage of aggregated collagen proportional area.

## Supplementary Materials

The following are available online at https://www.mdpi.com/2075-4418/10/11/895/s1, Figure S1: (A) SHG/TPEF image. (B) Illustrations of width, length, perimeter and area of a collagen fiber, Table S1. Intra-tumoral fibrosis in different groups classified by clinicopathological parameters, Table S2: PS fibrosis in different groups classified by clinicopathological parameters.

## Abbreviations:

ccRCC	clear cell renal cell carcinoma
SHG/TPEF	second harmonic generation/two-photon excitation fluorescence
PC	Pseudo-capsule
CPA	collagen proportional area
AggCPA	percentage of aggregated collagen proportional area
DisCPA	percentage of distributed collagen proportional area
