# Supplementary material for: The Significance of Fibrosis Quantification as a Marker in Assessing Pseudo-Capsule Status and Clear Cell Renal Cell Carcinoma Prognosis
Source: Diagnostics (Basel). 2020 Nov 2;10(11):895. doi: 10.3390/diagnostics10110895 (PMC7692106; doi:10.3390/diagnostics10110895)
Supplement: Supplementary file 1 [file diagnostics-10-00895-s001.pdf]

Supplementary Materials

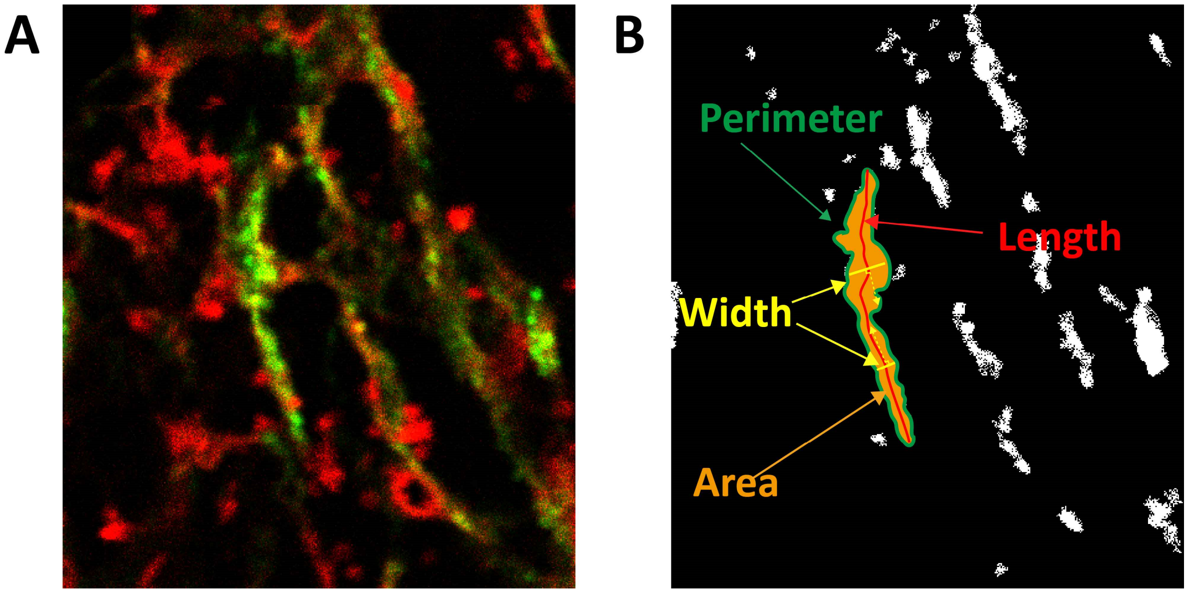

Figure S1 (A) SHG/TPEF image, (B) illustrations of width, length, perimeter and area of a collagen fiber.

Table S1. Intratumoral fibrosis in different groups classified by clinicopathological parameters

features	No.	CPA (Median±IQR)	P value	AggCPA (Median±IQR)	P value	DisCPA (Median±IQR)	P value
Gender							
Male	54	1.71±2.74	0.669	1.18±2.45	0.772	0.19±0.36	0.466
Female	19	1.12±2.20		0.80±1.67		0.17±0.34	
Age							
≤58	39	1.69±2.67	0.167	1.23±2.36	0.207	0.20±0.36	0.167
>58	34	1.26±2.93		0.79±2.55		0.17±0.36	
Fuhrman grade							
G1+2	54	1.82±2.74	0.615	1.20±2.44	0.563	0.18±0.33	0.624
G3+4	19	1.51±2.33		0.92±1.72		0.17±0.47	
Clinical Stage							
I+II	47	2.30±3.70	0.007	1.80±3.14	0.003	0.20±0.34	0.534
III+IV	26	1.05±1.76		0.56±1.13		0.15±0.37	
Tumor Size							
≤4cm	47	1.06±2.75	0.040	2.05±3.35	0.039	0.23+0.29	0.007
>4cm	26	2.35±3.02		3.24+5.41		0.37+0.29	
Histologic Necrosis							
Absent	54	1.23±2.73	0.227	0.92±2.42	0.274	0.14±0.35	0.030
Present	19	1.96±2.42		1.35±2.60		0.27±0.43	

Table S2. PS fibrosis in different groups classified by clinicopathological parameters

features	No.	CPA (Median±IQR)	P value	AggCPA (Median±IQR)	P value	DisCPA (Median±IQR)	P value
Gender							
Male	54	17.91±8.57	0.421	15.18±8.85	0.530	1.88±1.05	0.069
Female	19	16.50±14.68		14.29±12.85		1.46±0.79	
Age							
≤58	39	19.39±8.06	0.029	16.09±10.30	0.043	1.76±0.94	0.588

>58	34	16.50±9.98		13.61±9.84		1.68±1.20	
Fuhrman grade							
G1+2	54	17.50±12.19	0.990	14.71±11.92	0.753	1.52±0.88	0.004
G3+4	19	18.41±8.27		15.22±8.29		2.10±1.11	
Clinical Stage							
I+II	47	19.09±7.93	0.003	15.78±8.13	0.003	1.53±0.97	0.041
III+IV	26	14.13±9.51		10.81±8.55		1.98±0.90	
Tumor Size							
≤4cm	47	18.07±9.66	0.712	15.02±9.29	0.519	1.61±0.91	0.044
>4cm	26	17.24±10.31		14.50±10.72		2.00±0.94	
Histologic Necrosis							
Absent	54	17.70±10.12	0.860	14.87±9.86	0.725	1.61±0.86	0.159
Present	19	18.76±10.91		15.34±11.86		1.96±1.27	
